# Diagnosis of NLRP3 somatic mosaicism in CINCA/NOMID patients using next-generation sequencing

**DOI:** 10.1186/1546-0096-9-S1-P292

**Published:** 2011-09-14

**Authors:** K Izawa, R Nishikomori, N Tanikaze, MK Saito, R Goldbach-Mansky, I Aksentijevich, T Yasumi, T Kawai, T Nakahata, T Heike, O Ohara

**Affiliations:** 1 Department of Pediatrics, Kyoto University Graduate School of Medicine, Kyoto, Japan; 2 Translational Autoinflammatory Disease Section, Translational Autoinflammatory Disease Section NIH/NIAMS, Bethesda, Maryland, USA; 3 The National Institute of Arthritis and Musculoskeletal and Skin Diseases, Bethesda, Maryland, USA; 4 Department of Human Genome Research, Kazusa DNA Research Institute, Chiba, Japan

## Background

Chronic infantile neurological cutaneous and articular syndrome (CINCA), also known as neonatal-onset multisystem inflammatory disease (NOMID) is characterized by urticarial rash, neurological manifestations and arthropathy. This dominantly-inherited systemic autoinflammatory disease is provoked by heterozygous germline gain-of-function NLRP3 mutations, although conventional genetic analyses failed to detect disease-causing mutations in approximately 40% of patients. In these patients, NLRP3 somatic mosaicism was reported to be disease-causing and recently, high incidence of NLRP3 somatic mosaicism was reported in the international study. In the study, subcloning and Sanger sequencing were used to detect NLRP3 somatic mosaicism. However, the method requires time-consuming sample preparation and enormous cost to run enough sequencing per sample to detect low-level somatic mosaicism.

## Aim

To reduce the work and cost for detecting the low-level NLRP3 somatic mosaicism, we performed next-generation sequencing on “mutation-negative” CINCA/NOMID patients.

## Methods

Genomic DNA was isolated from previously reported Japanese CINCA/NOMID patients with NLRP3 somatic mosaicism, NLRP3 heterozygous mutations, healthy donors, and new “mutation-negative” CINCA/NOMID patients. All 9 NLRP3 exons were amplified by using specially designed primers in 2nd step PCR. We used the Roche GS-FLX 454 Genome Sequencer which had longer read length than other widely used next-generation sequencers.

## Results

NLRP3 somatic mosaicism was identified in all previously reported Japanese CINCA/NOMID patients with NLRP3 somatic mosaicism, and 4 out of the new 10 “mutation-negative” CINCA/NOMID patients. No mosaicism was detected in 50 healthy donors.

## Conclusions

Somatic mutations of NLRP3 can be identified by using next-generation sequencing with reduced workload and costs.

